# T193. CHRONIC HALOPERIDOL TREATMENT INDUCES SIGNIFICANT CHANGES IN MICROGLIA AND IN THE EXPRESSION OF THE 18 KDA TRANSLOCATOR PROTEIN TSPO IN NAIVE ADULT RAT BRAINS

**DOI:** 10.1093/schbul/sby016.469

**Published:** 2018-04-01

**Authors:** Marie Cotel, Ewelina Lenartowicz, Sridhar Natesan, Maria Dadabhoy, Anthony Vernon

**Affiliations:** 1 Institute of Psychiatry, Psychology & Neuroscience, King’s College London; 2 Research Center for Molecular Medicine of the Austrian Academy of Sciences

## Abstract

**Background:**

Positron emission tomography (PET) using radiolabeled ligands selective for the 18 kDa translocator protein (TSPO) is the most widely used technique to assess putative neuroimmune abnormalities in vivo. However, the results of TSPO PET in schizophrenia patients are ambiguous. In particular, the influence of antipsychotic medication exposure has not been definitively tested. We have previously established that chronic exposure to two different antipsychotics resulted in increased density and amoeboid morphology of brain microglia, particularly in the cortex (ACC) and corpus striatum (STR) (Cotel et al., 2015). Here we explore the consequences of such treatment on TSPO, in relation to changes in microglia. We hypothesize that chronic haloperidol treatment would increase the expression of TSPO in microglia and correlate with increased levels of circulating proinflammatory cytokines.

**Methods:**

Adult male Sprague-Dawley rats (12 week-old) were implanted with subcutaneous osmotic mini-pumps (ALZET® 2ML4) delivering a continuous dose of either common vehicle (20% β-cyclodextrin + 5% ascorbic acid) or haloperidol (2mg/kg/d) for 28 days (Kapur et al., 2003). Plasma was collected before perfusing the animals with 4% buffered PFA. Brains were carefully removed and processed for immunostaining against Iba1 and TSPO. Levels of plasma cytokines were measured using the pro-inflammatory 9-plex panel from Meso Scale Discovery (MSD®). TSPO expression was measured by its area fraction using FIJI software (10 snaps per animal per brain region, 20x magnification) in the ACC, STR, hippocampus (HPC), secondary somatosensory cortex (S2), primary motor cortex (M1). Numbers of resting and amoeboid microglia were determined by unbiased stereology (Gundersen, 1987) in the ACC, STR, and HPC. The Cavalieri estimator (StereoInvestigator 12.0,MBF Bioscience) was used to determine the volume of these regions. Data were analyzed by t-tests corrected for multiple comparisons, using GraphPad Prism 6.0.

**Results:**

Only 5 cytokines from the panel were above the limit of detection. Of these, IL-6 is significantly increased in haloperidol-treated animals (+186%, p<.01). IL-10, IL-4, KC/GRO, and TNFα plasma levels were not significantly affected. In parallel, TSPO expression was significantly increased in the ACC (+56%, p<.01), the STR (+77%, p<.01) and the S2 (+158%, p<.001). In the STR, the density of both resting and amoeboid microglia were significantly elevated in the treatment group (+22%, p<.05 and +45%, p<.001, respectively). A 2-way ANOVA revealed a significant interaction between treatment and brain region for amoeboid but not resting microglia. Peripheral IL-6 levels correlated negatively with TSPO levels in the STR in saline and haloperidol-treated groups (r=-0.82 and r=-0.62, respectively, p<.05). In addition, KC/GRO, although not different between groups, correlated positively with TSPO levels in STR in controls (r=0.77, p<.05), but negatively in HPC, M1, and S2 after haloperidol treatment (r=-0.66, r=-0.62, r=-0.6, respectively, p<.05).

**Discussion:**

As hypothesized, TSPO expression is significantly affected by chronic haloperidol, with augmented levels in the ACC, STR and S2. These data are in line with an increased TSPO PET signal in medicated schizophrenia patients as compared to matched antipsychotic-naïve patients (Holmes et al., 2016). Furthermore, these data confirm and extend our prior findings demonstrating that microglial activation is present already after 28 days of exposure to haloperidol and this effect is most prominent in the STR. Further investigations are needed to determine which cell types drive changes in TSPO (glia, endothelium, astrocytes) and how this relates to their functions at a molecular level.

